# Rapid detection of hepatitis C virus using recombinase polymerase amplification

**DOI:** 10.1371/journal.pone.0276582

**Published:** 2022-10-25

**Authors:** Catherine T. Chia, Andrew T. Bender, Lorraine Lillis, Benjamin P. Sullivan, Coleman D. Martin, Wynn Burke, Charles Landis, David S. Boyle, Jonathan D. Posner

**Affiliations:** 1 Department of Biochemistry, University of Washington, Seattle, Washington, United States of America; 2 Department of Mechanical Engineering, University of Washington, Seattle, Washington, United States of America; 3 PATH, Seattle, Washington, United States of America; 4 Department of Chemical Engineering, University of Washington, Seattle, Washington, United States of America; 5 Department of Medicine, Division of Gastroenterology, University of Washington, Seattle, Washington, United States of America; 6 Department of Laboratory Medicine and Pathology, University of Washington, Seattle, Washington, United States of America; 7 Family Medicine, School of Medicine, University of Washington, Seattle, Washington, United States of America; Kyoto University, JAPAN

## Abstract

Over 71 million people are infected with hepatitis C virus (HCV) worldwide, and approximately 400,000 global deaths result from complications of untreated chronic HCV. Pan-genomic direct-acting antivirals (DAAs) have recently become widely available and feature high cure rates in less than 12 weeks of treatment. The rollout of DAAs is reliant on diagnostic tests for HCV RNA to identify eligible patients with viremic HCV infections. Current PCR-based HCV RNA assays are restricted to well-resourced central laboratories, and there remains a prevailing clinical need for expanded access to decentralized HCV RNA testing to provide rapid chronic HCV diagnosis and linkage to DAAs in outpatient clinics. This paper reports a rapid, highly accurate, and minimally instrumented assay for HCV RNA detection using reverse transcription recombinase polymerase amplification (RT-RPA). The assay detects all HCV genotypes with a limit of detection of 25 copies per reaction for genotype 1, the most prevalent in the United States and worldwide. The clinical sensitivity and specificity of the RT-RPA assay were both 100% when evaluated using 78 diverse clinical serum specimens. The accuracy, short runtime, and low heating demands of RT-RPA may enable implementation in a point-of-care HCV test to expand global access to effective treatment via rapid chronic HCV diagnosis.

## Introduction

More than 71 million people are infected with hepatitis C virus (HCV) worldwide, but chronic HCV infections are frequently asymptomatic and thus 80% of those infected are unaware of their illness [1]. The limited availability of HCV testing has led to under-diagnosed and under-treated chronic HCV infections [2]. Untreated HCV infection may lead to chronic liver disease and hepatocellular carcinoma, accounting for an estimated 400,000 annual global deaths [1]. Within the last six years, direct-acting antivirals (DAAs) have demonstrated high cure rates (90–95%) across all HCV genotypes in under 12 weeks of all-oral treatment [3]. These highly effective therapeutics offer a path toward ending the HCV epidemic as these drugs become more affordable; however, the successful rollout of DAAs relies on accurate and timely diagnostic testing to identify cases of HCV viremia and link eligible patients to DAA treatment [4].

The clinical standard of care for HCV testing is to use a rapid antibody test for screening, followed by a nucleic acid amplification test (NAAT) for HCV RNA to confirm active viremia before initiating DAA treatment. Following completion of the DAA regimen, another NAAT is needed to confirm HCV cure, which is indicated by a sustained virological response after 12 weeks post-treatment (SVR12) [4, 5]. Current PCR-based HCV RNA testing requires well-instrumented, centralized laboratories due to cold chain dependent reagents, delicate instrumentation, reliable electrical power, proficient laboratory staff, and appropriate infrastructure, to host required equipment [6]. In outpatient clinics, the current testing protocol can result in delayed diagnoses that prevent immediate linkage to DAA treatment or complete loss-to-follow-up, especially in low and middle-income countries (LMICs) [7]. Recent initiatives have encouraged the development of rapid, clinic-based HCV RNA tests to provide an accessible diagnosis of chronic HCV [4, 5]. Decentralized HCV RNA testing offers a streamlined approach to patient care where a rapid NAAT may be used to diagnose HCV, identify active viremia, and link patients to DAAs during a single clinic visit.

PCR-based testing platforms for rapid HCV RNA detection must address the inherent challenges of thermocycling, which typically takes 1–2 hours to generate a result and requires device hardware for heating and precisely controlling reaction temperatures. In the last two decades, several isothermal nucleic acid amplification methods have been developed to amplify nucleic acids at a single reaction temperature, amplify in the presence of common amplification inhibitors, and return results in significantly less time than traditional PCR [8–12]. Isothermal amplification assays enable new point-of-care (POC) testing capabilities, such as simple and rapid benchtop NAAT assays that may be performed in low-complexity clinic-based labs or fully integrated POC tests for either clinical or home settings. The most widely studied of these methods is loop-mediated isothermal amplification (LAMP), which offers high analytical sensitivity and low-cost reagents that are widely available [10]. LAMP requires an incubation temperature of ~65°C and runtimes of 40–60 minutes but has issues with false-positive results when common visual or intercalating dye detection methods are employed [13–15]. Recombinase polymerase amplification (RPA) has emerged as another leading isothermal amplification method that addresses the key shortcomings of LAMP. RPA employs recombination and single-stranded binding proteins to facilitate primer insertion for rapid DNA amplification, and fluorescence detection is easily integrated with a sequence-specific probe that is cleaved by an exonuclease upon hybridization to an amplicon [11]. RPA features a rapid time-to-results (10–15 minutes), low operating temperature (35–42°C), and analytical sensitivity equivalent to PCR [11, 16]. These features enable RPA implementation in POC assays for pathogen detection by significantly reducing time-to-results for the patient and alleviating the electrical power demands for heating reactions [17].

In reviewing the literature, we found several studies describing reverse transcription LAMP (RT-LAMP) assays to detect HCV [18–23]. These studies have shown RT-LAMP can detect all six HCV genotypes, provide high sensitivity in testing clinical samples, and offer several useful detection mechanisms. There are several key limitations of RT-LAMP including runtimes of up to 60 minutes and poor clinical specificity due to the lack of a target-specific detection method [18–20]. Despite significant advancements in RT-LAMP for HCV detection, we are unaware of any published studies using other isothermal amplification assays for HCV that feature significantly shorter runtimes and lower incubation temperatures, such as RPA.

In this paper we describe the development of a reverse transcription RPA (RT-RPA) assay for HCV detection that features a rapid runtime, minimal instrumentation, pan-genomic detection, and high sensitivity and specificity when evaluated with diverse clinical samples. The RT-RPA primer and probe sequences were designed to target a highly conserved region of the HCV genome. The limit of detection of the RPA assay was measured using HCV genotype 1 RNA standards, as well as purified viral RNA from genotypes 1–6. We evaluated the clinical sensitivity and specificity of the assay using 78 clinical serum specimens. This work provides key advantages over LAMP-based HCV RNA detection assays due to its speed, high diagnostic performance, and reduced power demands due to its low operating temperature. There is a clear clinical need for improved rapid assays for chronic HCV that are appropriate for point-of-care applications, which is highlighted in a recent minireview article outlining the need for an RT-RPA assay for HCV [24]. RT-RPA may play a key role in expanding access to decentralized chronic HCV testing and linking HCV-positive patients to life-saving medication.

## Materials and methods

### Clinical specimens and nucleic acid extraction

A total of 78 clinical specimens were used for the clinical sensitivity and specificity analysis of the RPA assay. 58 anonymized HCV-positive human serum specimens were selected randomly from the biorepository at the Gastrointestinal Center for Analytical Research and Exploratory Science (GiCasRes) at the University of Washington. All specimens were originally collected from patients at the Hepatitis & Liver Clinic at Harborview Medical Center (Seattle, USA) under ethical approval granted by the institutional review board at the University of Washington. All samples were obtained with written informed consent. At the time of collection, the specimens were confirmed HCV-positive using the Abbott *RealTime* HCV Assay and genotyped with the Abbott *RealTime* HCV Genotype Assay (Abbott Laboratories, Abbott Park, USA). An additional 20 HCV-negative serum samples from individual donors were obtained from BioIVT (Hicksville, NY). All samples were stored at -80°C until use.

The QIAamp Viral RNA Mini Kit (Qiagen, Hilden, Germany) was used to extract nucleic acids from clinical samples, following the manufacturer’s protocol. The input sample volume was 140 μL of serum or plasma, and the nucleic acids were removed from the column using a double elution of 40 μL each, totaling 80 μL of purified RNA for each sample. The eluted RNA was then aliquoted and stored at -80°C.

### Synthesis of RNA standards

HCV RNA standards were synthesized using double-stranded DNA gene fragments (Integrated DNA Technologies, Coralville, USA) containing the complete 5’UTR gene and a 5’-T7 RNA polymerase promoter sequence. The 5’UTR sequence was from the NCBI reference sequence for HCV genotype 1 (accession number NC_004102) [25]. The lyophilized gene fragments were rehydrated and stored at -20°C. The DNA fragments were transcribed into RNA with the MEGAscript T7 Transcription Kit following the manufacturer’s protocol including the DNase treatment step (Thermo Fisher Scientific, Waltham, USA). The RNA transcripts were purified using the MEGAclear Transcription Clean-Up Kit (Thermo Fisher Scientific) and stored at -80°C. RNA transcripts were then quantified using the Quant-iT RNA Assay Kit (Thermo Fisher Scientific, Waltham, USA) on a microplate reader (SpectraMax iD3, Molecular Devices, San Jose, USA).

### RT-RPA assay details

The RT-RPA HCV detection assay consists of lyophilized pellets of the TwistAmp Exo RPA reaction kits (TwistDx Ltd., Maidenhead, United Kingdom) with the addition of a master-mix for a total volume of 50 μL per RPA reaction. The master-mix added to each reaction tube includes TwistAmp rehydration buffer (29.5 μL), forward primer (2.1 μL of 100 μM stock solution), reverse primer (2.1 μL), FAM-labeled probe (0.6 μL), and DEPC-treated water (Integrated DNA Technologies, see S1 Table for primer sequences). The one-step RT-RPA protocol included 2.5 μL of target RNA and 1 μL of 200 U/μL SuperScript reverse transcriptase (Thermo Fisher Scientific). The two-step RT-RPA experiments included 2.5 μL of cDNA and no reverse transcriptase. Following the manufacturer’s instructions, 2.5 μL of 280 mM magnesium acetate was added to the caps of individual reaction tubes, and tubes were manually agitated before being placed into a T16-ISO instrument (Axxin, Fairfield, Australia) for incubation and real-time fluorescence measurement. The tubes were incubated at 39°C for a total time of 17 minutes, with a brief manual mixing step after 4 minutes. Data was analyzed and plotted using MATLAB (Mathworks, Natick, USA). All RPA data was background subtracted using the fluorescence value at the 5-minute mark, directly after mixing the reaction. The fluorescence threshold used to determine positive versus negative results was 50 arbitrary fluorescence units.

Experiments that evaluated the RT-RPA assay’s limit of detection for HCV RNA standards were performed using a one-step RT-RPA protocol, where RNA transcripts were spiked directly into RT-RPA reactions. All other data reported in this work used a two-step RT-RPA protocol with a separate reverse transcription step. The SuperScript IV First-Strand Synthesis Kit (Thermo Fisher Scientific, Waltham, USA) was used for complementary DNA (cDNA) synthesis from extracted viral RNA following the manufacturer’s protocol. The volume of input RNA was 10 μL (20 μL total reaction volume), and random hexamers at a final concentration of 2.5 μM were used to initiate first-strand synthesis.

### RT-qPCR assay

Two-step quantitative reverse transcription PCR (RT-qPCR) was used to measure the viral loads of the clinical specimens. The RT-qPCR primers and thermal profile were originally developed by Roche Molecular Systems for their COBAS AMPLICOR and TaqMan HCV tests [26, 27]. First-strand synthesis was first performed using the SuperScript IV kit, followed by amplification using the TaqMan Fast Advanced Master Mix reaction (Thermo Fisher Scientific). Each 20 μL reaction contained 10 μL master mix, 0.9 μL of 10 μM forward primer, 0.9 μL of 10 μM reverse primer, 0.25 μL of 10 μM probe, and 2.5 μL of cDNA. Amplification was conducted in 96 well plates on an Applied Biosystems QuantStudio 3 Real-Time PCR System (Thermo Fisher Scientific) using the following thermal cycling profile: 95°C for 1:30 minutes, followed by 40 amplification cycles of 10 s at 95°C and 45 s at 58°C.

Viral loads of clinical samples were quantified by calculating copy numbers from a standard curve generated from quantified 5’ UTR cDNA using an established method [28]. Experimental data was processed with the Standard Curve Analysis Module of the QuantStudio Design and Analysis Software (Thermo Fisher Scientific). Separate standard curves were generated for each PCR-plate run, and all standard curve concentrations and unknown samples were run in triplicate.

### Limit of detection and analytical specificity analysis

To evaluate the limit of detection (LOD) of the RPA assay for each respective genotype, viral RNA from quantified and genotyped clinical samples (GiCasRes biorepository) were reverse transcribed and spiked into RPA at varying concentrations. The samples representing genotype 5 and genotype 6 were not available through the GiCasRes biorepository and were instead obtained from SeraCare (Milford, USA). The analytical specificity of the RPA HCV assay was evaluated by evaluating the reactivity of the assay on extracted viral RNA from HIV-1, hepatitis A virus, West Nile virus, dengue virus, influenza A virus, zika virus, and MS2 bacteriophage. These purified viral RNAs were obtained from BEI Resources (NIAID, NIH), reverse transcribed into cDNA, and spiked into RPA at 1000 cp/rxn using the protocol described previously.

### Clinical sensitivity and specificity analysis

The clinical sensitivity and specificity were evaluated using 58 clinical specimens (GiCasRes biorepository) that were confirmed HCV-positive and 20 human serum samples confirmed HCV-negative via RT-qPCR. Extracts from clinical samples were reverse transcribed into cDNA and spiked directly into RPA for qualitative analysis.

## Results

### RPA assay overview and design

**Fig 1:**
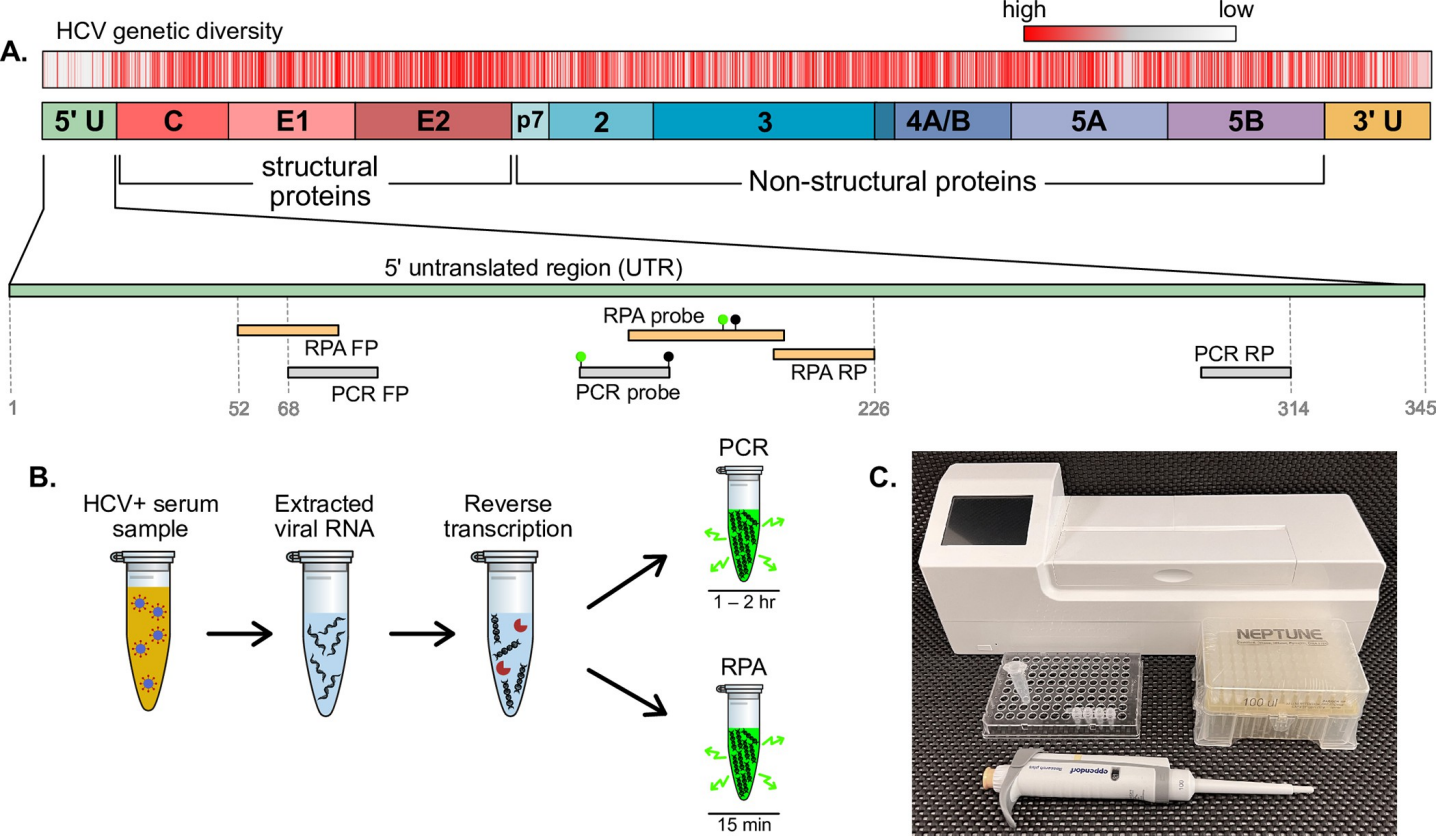
RT-RPA target sequence location and overview of assay. (A) HCV RPA assay was designed to target the 5’ untranslated region (UTR) gene which has the highest homology across all genotypes. We plot locations of mismatches in red for an alignment of the reference sequences of the six genotypes of HCV, with regions of high homology colored grey or white. We show the locations of the RPA primers and probe on 5’ UTR, as well as the published PCR primers and probe [26]. (B) Schematic of the experimental protocol for HCV RNA detection using either RT-RPA or RT-PCR. Viral RNA is extracted from human serum using a column-based solid phase extraction kit, RNA is reverse transcribed into complementary DNA (cDNA), and HCV cDNA is detected with either PCR (1–2 hr runtime) or RPA (15 min runtime). (C) A simple experimental setup for reverse transcription and RPA includes basic laboratory consumables, a pipette, and a benchtop fluorometer with an integrated heating block (Axxin T16).

The oligonucleotide sequences for the RT-RPA primers and probe were designed to target the most conserved region of the HCV genome across the six genotypes. An alignment of the National Center for Biotechnology Information (NCBI) reference sequences of HCV genotypes 1–6 using the NCBI Multiple Sequence Alignment tool identified the highest sequence homology in the 5’ untranslated region (5’ UTR), as seen in Fig 1A. Candidate RPA primers and probes were designed to target conserved regions of 5’ UTR, and primers were screened based on previous experience designing RPA assays for HIV and primer selection recommendations from the original manufacturer of RPA reagents [29–31]. The best performing combination of primers and probe was selected based on the greatest increase in fluorescence signal and the least time to reach the fluorescence threshold when amplifying HCV RNA standards. The locations of the final RPA primer and probe sequences are shown in Fig 1A with respect to the base number of the HCV genotype 1 sequence (accession number NC004102). Fig 1A also identifies the target regions of the PCR primers and probe that were used as the gold standard reference assay in this work. This RT-PCR assay was originally developed by Roche Molecular Systems for their COBAS AMPLICOR and TaqMan HCV tests [26, 27]. All primer and probe sequences used in this work can be found in S1 Table.

The protocol for detecting HCV from clinical samples is illustrated in Fig 1B. Viral RNA was extracted from serum samples using a commercial kit for column-based solid phase extraction. Extracted RNA was reverse transcribed into cDNA, followed by amplification and fluorescence-based detection using RPA and a gold-standard PCR assay for comparison. The RPA assay has a runtime of 17 minutes while PCR takes 1 to 2 hours to run, depending on the thermal cycling capability of the PCR machine. The instrumentation for reverse transcription and RPA is relatively simple, requiring only a pipette and benchtop fluorometer that is specifically designed for measuring isothermal amplification reactions in a field-deployable format (Fig 1C).

### Limit of detection and analytical specificity

**Fig 2:**
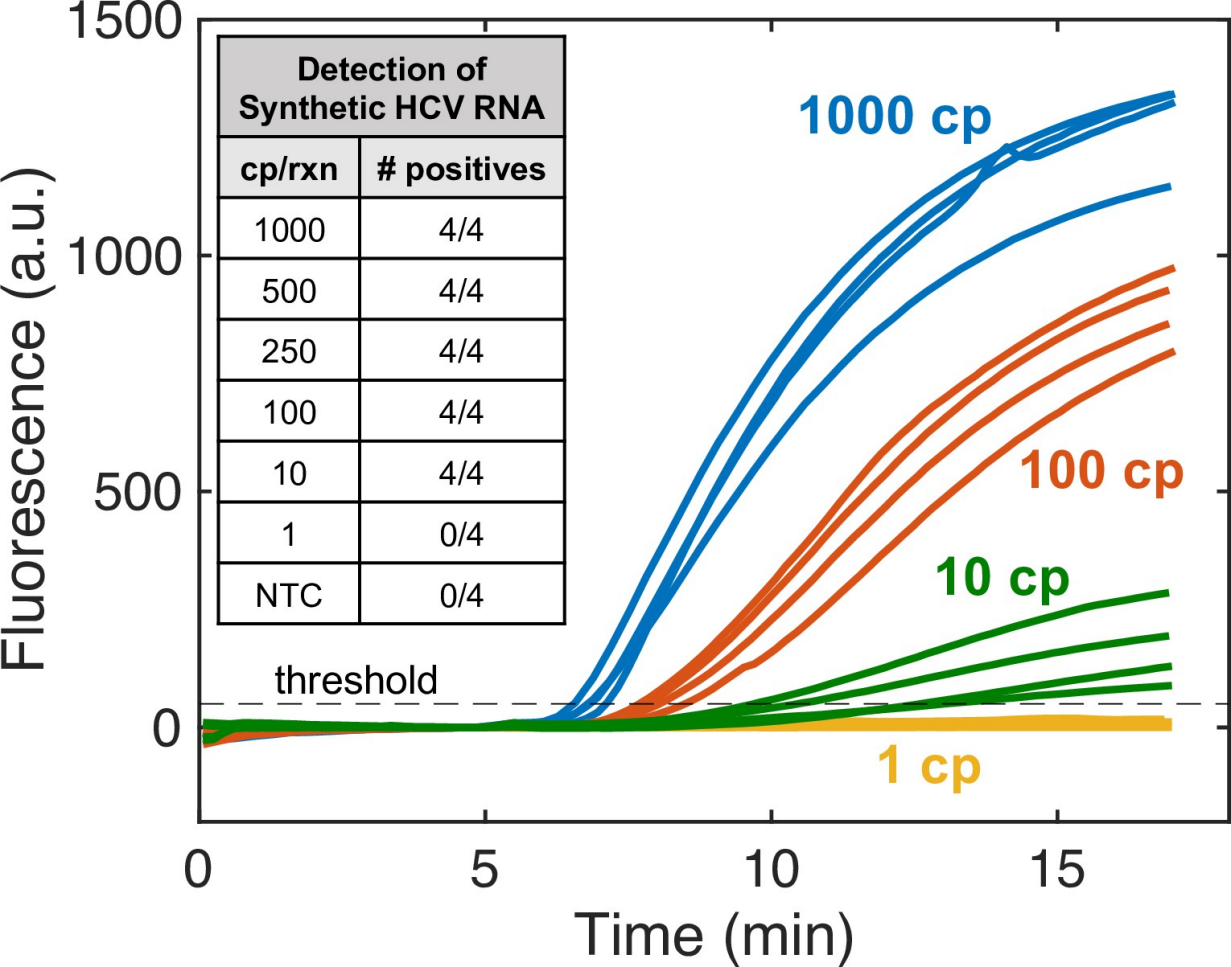
Detection limit of HCV RNA standards. The limit of detection of one-step RT-RPA was measured using HCV RNA synthetic standards. Real-time fluorescence measured from reaction tubes is plotted over 17-minutes for experimental data of 1000, 100, 10, and 1 copy per reaction. All reactions with input copies of RNA greater than or equal to 10 copies per reaction amplified in 15-minutes or less, while replicates with 1 copy per reaction and no template controls (NTCs) did not amplify. The inset table lists data for all input RNA concentrations tested. All concentrations were run with 4 replicates.

The limit of detection (LOD) of the RPA assay was first investigated using HCV RNA standards created via *in vitro* transcription from a DNA gene fragment of 5’ UTR. Quantified RNA transcripts were spiked into RPA at known concentrations to evaluate the LOD. These experiments employed a one-step RT-RPA protocol with reverse transcriptase included in the RPA reaction mix. Real-time fluorescence monitoring of the reactions resulted in the amplification curves plotted in Fig 2, with positive reactions passing the fluorescence threshold. Varying input copy numbers of HCV RNA transcripts were added to reactions to determine the LOD of the assay, summarized in the Fig 2 inset. The LOD of the RT-RPA assay using HCV RNA transcripts was 10 copies per reaction (cps/rxn). All positive reactions could be identified within 15 minutes, and reactions with 100 input copies or greater amplified over the fluorescence threshold within 8.5 minutes. RT-RPA could not detect RNA at 1 cp/rxn, and the no template controls (NTCs) also did not amplify. Control experiments with 1000 cps/rxn of RNA and no reverse transcriptase were performed, confirming that there were no residual DNA gene fragments in the RNA standards used (see S1 Fig).

The performance of the RT-RPA assay was also evaluated using viral HCV RNA from genotypes 1–6 that were extracted from human serum samples. In contrast to HCV RNA standards synthesized from the 5’ UTR gene, extracted viral RNA exhibited inconsistent amplification with one-step RT-RPA. However, two-step RT-RPA featured reliable and sensitive amplification of extracted HCV RNA. Table 1 shows that the two-step RT-RPA assay detects all six HCV genotypes, although the performance differs marginally across genotypes. Genotype 1 has the lowest LOD of 25 cps/rxn. Genotypes 2, 4, and 5 all have LODs less than or equal to 100 cps/rxn, while genotypes 3 and 6 were consistently detected at 500 and 250 cps/rxn, respectively. There were no amplification events observed in the no template control (NTC) reactions performed in this analysis.

**Table 1:** Pangenomic HCV RNA detection. Viral RNA from the six HCV genotypes at various input copy numbers were amplified via two-step RT-RPA. Genotyped viral RNA was extracted from HCV-positive human serum and spiked into reverse transcription and RPA reactions for detection. The lowest LODs were observed for genotypes 1 and 4. Genotypes 3 and 6 were the most challenging to detect with inconsistent amplification observed at 100 copies per reaction or less. All no template controls (NTCs) did not amplify in RPA (N = 28).

HCV Genotype	cp/rxn	# positives
**1**	500	4/4
	100	4/4
	50	4/4
	25	4/4
	10	1/4
**2**	500	4/4
	100	4/4
	25	3/4
	10	0/4
**3**	500	4/4
	100	3/4
	25	0/4
**4**	75	6/6
	15	5/6
**5**	500	4/4
	100	4/4
	50	0/4
**6**	500	4/4
	250	4/4
	100	0/4
**negative**	0	0/28

The analytical specificity of the two-step HCV RT-RPA assay was screened using a range of viral RNA from bloodborne viruses to ensure that foreign viral nucleic acid does not result in cross reactivity in the assay due to non-specific primer or probe binding. The bloodborne viruses screened in this work were selected based on their worldwide prevalence, and while not comprehensive, they provide a preliminary analytical specificity confirmation. The RPA assay was screened for cross reactivity against several other common viruses present in clinical laboratories: SARS-CoV-2, influenza A, and MS2 bacteriophage–a common internal amplification control used in commercial assays. As shown in Table 2, the RPA assay did not amplify in the presence of any foreign nucleic acid from common viruses.

**Table 2:** Preliminary analytical specificity screening. Reactivity of RT-RPA to viral RNA from common bloodborne viruses and widely circulating respiratory viruses. RPA reactions were performed with 1000 cps/rxn for all viral nucleic acids.

Viral RNA	# positives
Hepatitis C virus	3/3
HIV-1	0/3
Hepatitis A virus	0/3
West Nile virus	0/3
Dengue virus	0/3
Zika virus	0/3
MS2 phage	0/3
SARS-CoV-2	0/3
Influenza A	0/3

### Clinical sensitivity and specificity

The clinical utility of the two-step RT-RPA assay was evaluated by testing clinical specimens from HCV-positive and HCV-negative individuals. The clinical sensitivity and specificity of RPA for HCV detection was measured using an RT-PCR assay as the reference standard. The RT-PCR testing was performed in-house, and this assay has been validated in previous publications and currently is used in a commercial HCV test from Roche [26, 27]. As presented in Table 3, the RT-RPA assay detected 58/58 HCV-positive serum samples and correctly identified 20/20 HCV-negative samples. Therefore, the clinical sensitivity and specificity were both 100%.

**Table 3:** Clinical sensitivity and specificity. Sensitivity and specificity of two-step RT-RPA were evaluated using clinical human serum samples that were HCV-positive or HCV-negative. Samples were confirmed positive or negative with a gold-standard PCR test.

		RPA Assay Result	
		Positive	Negative
**PCR Assay Result**	Positive	58	0
	Negative	0	20
	**Sensitivity**		100%
	**Specificity**		100%

**Fig 3:**
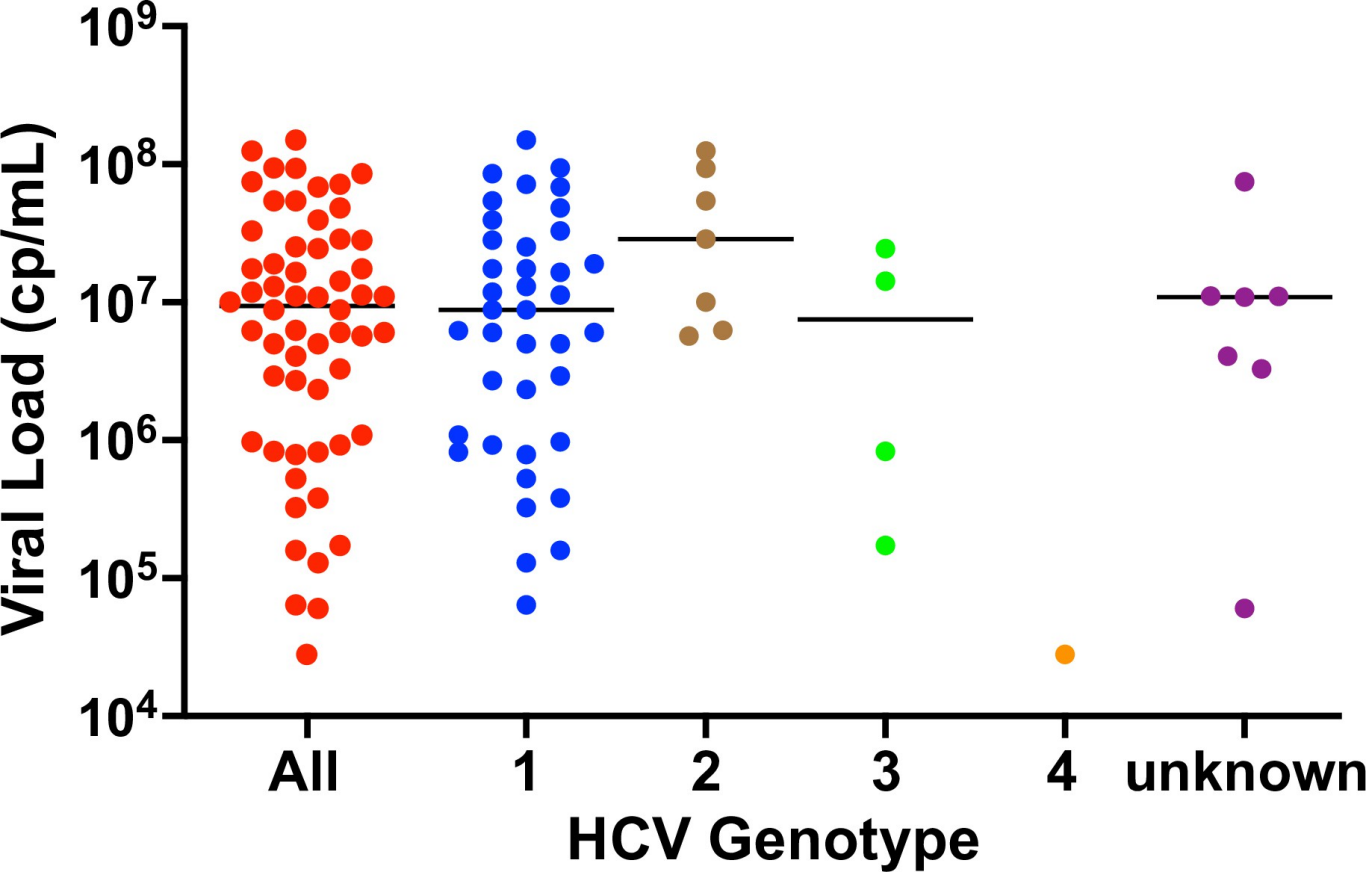
Viral load distribution of HCV clinical samples. Viral loads of clinical samples used in this study were measured using RT-qPCR on freshly extracted RNA from serum samples (GiCasRes biorepository). The measurements are stratified by genotype on the x-axis, and the black horizontal bar represents the median. We evaluated a total of 58 HCV-positive samples representing genotypes 1–4, and 7 samples lacking a paired genotype test.

The HCV-positive clinical samples were genotyped and quantified with RT-PCR to characterize the diversity of samples. Fig 3 plots the viral load distribution of the samples, stratified by genotype. There were 58 total HCV-positives samples with 7 samples with unknown genotypes, either through data loss in the biorepository or an inconclusive result from the genotyping assay in the clinical laboratory. The samples with known genotypes had a distribution of 76.5% (39/51) genotype 1, 13.7% (7/51) genotype 2, 7.8% (4/51) genotype 3, and 2.0% (1/51) genotype 4. The mean viral load of the samples was 7.36 log_10_ cps/mL, and the median was 6.97 log_10_ cps/mL.

## Discussion

We report on the development of an RT-RPA assay for detecting the six HCV genotypes, and we evaluate the performance of the assay with quantified RNA standards and clinical samples. The assay was designed to target the 5’UTR region of the HCV genome due to its high sequence homology. We observed rapid time-to-results with all RT-RPA reactions that were characterized as positives amplifying above the fluorescence threshold within 15 minutes, and some positive samples were detected as early as 6.5 minutes, as seen in Fig 2. This is significantly faster than PCR-based HCV assays, such as the one used in this work which has a runtime of 1.25 hours [26, 32, 33]. The 17-minute RT-RPA runtime is also a significant improvement over current RT-LAMP HCV assays which have runtimes ranging from 40 minutes to 1 hour [18–20]. The RT-RPA assay uses a sequence-specific probe that emits fluorescence only upon hybridizing to the amplified 5’UTR sequence. This is an advantage over common fluorescence and colorimetric detection strategies for LAMP which struggle with false positives due to nonspecific amplification [19, 34]. For example, a recent publication on RT-LAMP for HCV detection reported a clinical specificity of 91% and required an exact assay cutoff time to exclude false positives that occurred if incubation continued further [19]. The RT-RPA HCV assay demonstrated 100% clinical specificity, and no false positives were observed with the RPA assay even if incubated for twice the duration of the standard runtime (see S2 Fig).

The two-step RT-RPA assay features sensitive detection of the six HCV genotypes. Since HCV genotype 1 (GT1) is the most prevalent in the US and worldwide [35, 36], primer and probe sequences were designed to feature the fewest mismatches for GT1 (0 mismatches for HCV GT1, NC004102), resulting in higher numbers of mismatches for other genotypes. Across the primer and probe hybridization regions, there were 7 total mismatches for GT2, 13 for GT3, 6 for GT4, 3 for GT5, and 3 for GT6 (see S3 Fig and S2 Table). It is important to consider that these primer and probe mismatches were based on alignments with the NCBI reference sequences for each respective genotype. However, HCV has a high degree of genetic diversity with numerous subtypes and recombinant forms that complicate mismatch calculations among isolates within the same genotype [37]. Some studies have observed that RPA is uniquely capable of tolerating high numbers of target region mismatches with only a moderate reduction in assay sensitivity due to its longer primers [31, 38]. The lowest LOD for the RPA assay was observed for GT1 (25 cp/rxn), and the highest LOD was observed for GT3 (500 cp/rxn), which suggests the number of expected mismatches influences the assay sensitivity. The LOD for specific genotypes may be improved in future work by further optimizing the primer designs and/or introducing degenerate bases at common mismatch locations. However, as currently designed, RPA exhibits 100% clinical sensitivity and specificity for HCV detection with clinical samples representing genotypes 1–4.

The 58 HCV-positive specimens used in this study are a random collection of samples that the GiCasRes biorepository was able to provide. The genotype distribution of the samples was 76.5% (39/51) GT1, 13.7% (7/51) GT2, 7.8% (4/51) GT3, and 2.0% (1/51) GT4, which closely mirrors the genotype distribution reported for the United States. The US genotype distribution is approximately 75.4% GT1, 12.6% GT2, 10.2% GT3, 1.5% GT4, and 0.3% GT5/6, and the global distribution is approximately 46% GT1, 13% GT2, 22% GT3, 13% GT4, 1% GT5, 2% GT6, and 3% undefined genotypes [35, 36]. The median viral load of the HCV samples in this work (6.97 log_10_ cp/mL) was similar to the median viral load of 6.3 log_10_ IU/mL that was reported in a survey of HCV-positive individuals across 20 clinical trials with enrolled patients from 36 different countries spread across the Americas, Asia, Europe, Africa, and Australia [39]. In this work, viral loads and RNA concentrations are described in terms of copies rather than international units (IU) due to the method of quantification of the RNA standards used in RT-qPCR. As a rough approximation, Roche provides a conversion of 1 IU = 2.7 RNA copies for their HCV RT-qPCR assay, which is used in a slightly modified form in this work [40].

Initial LOD experiments used RNA standards created using *in vitro* transcription of a DNA gene fragment of 5’UTR. RNA transcripts are widely used in assay development for RNA standards or positive controls due in part to their ease of laboratory-based synthesis and quantification [28, 41]. RT-RPA featured highly sensitive detection of HCV RNA transcripts (10 cp/rxn) using a one-step reaction format. Surprisingly, HCV RNA extracted from clinical samples could not be amplified consistently with one-step RT-RPA and required a separate reverse transcription step with random hexamers to complete first strand synthesis. Separate reverse transcription prior to amplification is common in PCR protocols, and many of the published RT-PCR assays for HCV detection use a two-step approach [42–45]. It is unclear why extracted viral RNA is more challenging to reverse transcribe than RNA transcripts of the 5’UTR gene, although we suspect it may be due to persistent secondary structures that have been observed in full-length HCV RNA due to its high GC content [32, 46, 47]. Similar difficulties of this magnitude with reverse transcription of viral RNA in one-step RT-RPA reactions have not been reported in the literature or observed with other RT-RPA assays developed in our laboratories, including assays for HIV, SARS-CoV-2, and MS2 bacteriophage [30, 31, 48]. Although our preliminary efforts to optimize one-step RT-RPA for detecting extracted HCV RNA resulted in inconsistent performance, there is an ongoing expanded investigation of experimental conditions to improve the performance of single-pot reverse transcription and RPA. These efforts are focused on overcoming the strong secondary structure of HCV RNA that may prevent efficient reverse transcription. Potential strategies include leveraging higher RT-RPA incubation temperatures (39°C—48°C), different reverse primer binding sites, reaction additives to reduce secondary structures, and different reverse transcriptases.

## Conclusions

In this paper, we report on a rapid RT-RPA assay for detecting HCV RNA with high sensitivity and specificity. The isothermal nucleic acid amplification assay runs in 17 minutes at a single low incubation temperature of 39°C, requiring only a simple benchtop fluorometer for instrumentation. All genotypes of HCV were reliably detected with two-step RT-RPA, and an LOD of 25 cps/rxn was observed for viral RNA of genotype 1, the most prevalent strain. The assay demonstrated a clinical sensitivity of 100% and specificity of 100%, with no cross-reactivity to other common bloodborne viruses. This work has high specificity due to an amplicon sequence-specific fluorescent probe. Visual detection of RPA via lateral flow strips is readily enabled using the same probe design, a biotinylated primer, and inclusion of endonuclease IV in the reaction mix [30]. To our knowledge, this work is the first reported RPA assay for HCV. A recent publication leveraged two-stage RPA and RT-LAMP for HCV RNA detection, but that is distinct from standalone RPA [49]. RT-RPA offers several advantages over existing RT-LAMP HCV assays, notably shorter runtime and improved specificity. The per-test cost of RPA reagents is similar to that of PCR and LAMP (see S3 Table), but in the assay development stage, it is difficult to predict the ultimate per-test cost of a commercialized diagnostic assay, which is a function of intellectual property licensing fees, economies of scale, and other industry forces.

Future work is focused on improving the analytical sensitivity of one-step RT-RPA for viral HCV RNA detection and implementing the assay for decentralized chronic HCV testing. One potential approach for point-of-care testing is a sample-to-answer diagnostic device that integrates on-board sample preparation of blood with RT-RPA [50–53]. Similar POC nucleic acid tests have been developed for SARS-CoV-2 detection with protocols simple enough for at-home use (e.g. Lucira CHECK IT). Another potential decentralized testing strategy is to pair the HCV RT-RPA assay with a simple benchtop sample preparation method to create a stream-lined diagnostic protocol that can be performed in low-complexity laboratories in clinical settings by nurses or other technical staff. Several studies have demonstrated the feasibility for similar field-deployable RT-RPA assays using a laboratory-in-a-suitcase and simple user protocol for viral detection [54–56].

## Supporting information

S1 Table Sequences of the PCR primers, PCR probe, RPA primers, and RPA probe. nt = nucleotide; FAM = fluorescein amidite; [ZEN] = internal ZEN dark quencher, [3IABkFQ] = 3’ Iowa Black FQ, [dSpacer] = abasic site; [T(BHQ-1)] = Black Hole Quencher-1 bound to an internal thymine residue; Spacer C3 = a moiety that replaces the 3’-OH group preventing nucleotide extension from an intact probe.
(DOCX)

S2 Table Sequence mismatches between the RPA primers and probe with respect to each HCV genotype. As in S2 Fig, this uses the NCBI reference sequences for each genotype. This information is an estimate of mismatches due to random mutations, numerous subtypes, and recombinant forms that contribute to HCV genetic variability.
(DOCX)

S3 Table Per-test costs of commercially available reagents for RPA, LAMP, and PCR. (DOCX)

S1 Fig Control experiments with no reverse transcriptase for the one-step RT-RPA HCV assay. Data in blue represent RPA reactions that included both 250 copies of HCV RNA transcripts and 200 U of reverse transcriptase (N = 2). Data in red represent reactions with 250 copies of RNA transcripts but with no reverse transcriptase (N = 2). This demonstrates reverse transcriptase is needed for successful amplification, and that there are no contaminating DNA gene fragments of the HCV 5’UTR gene present in the RNA transcripts.
(TIF)

S2 Fig Extended incubation of one-step RT-RPA HCV assay. Experiments with extended incubation of RT-RPA demonstrate no template controls (NTCs) do not increase in fluorescence if incubated up to 40 minutes. Data in blue represent RPA reactions that included 250 copies of HCV RNA transcripts (N = 2), and data in red represent NTC reactions (N = 2).
(TIF)

S3 Fig Multiple sequence alignment of all six HCV genotypes and the RPA primers and probe. The NCBI reference sequences for each respective genotype were used here (accession numbers included in the label). Only the target region of the RPA assay is shown. Mismatches are highlighted in red with respect to the genotype 1 sequence.
(TIF)
