# Supplementary material for: Rapid detection of hepatitis C virus using recombinase polymerase amplification
Source: PLoS One. 2022 Oct 25;17(10):e0276582. doi: 10.1371/journal.pone.0276582 (PMC9595512; doi:10.1371/journal.pone.0276582)
Supplement: S1 Table — nt = nucleotide; FAM = fluorescein amidite; [ZEN] = internal ZEN dark quencher, [3IABkFQ] = 3’ Iowa Black FQ, [dSpacer] = abasic site; [T(BHQ-1)] = Black Hole Quencher-1 bound to an internal thymine residue; Spacer C3 = a moiety that replaces the 3’-OH group preventing nucleotide extension from an intact probe. (DOCX) [file pone.0276582.s001.docx]

| **Description** | **Sequence (5’ – 3’)** |
| --- | --- |
| PCR forward primer  (24 nt) | GCAGAAAGCGTCTAGCCATGGCGT |
| PCR reverse primer  (24 nt) | CTCGCAAGCACCCTATCAGGCAGT |
| PCR probe  (20 nt) | [56-FAM]-CATAGTGGT[ZEN]CTGCGGAACCGGTGAGT[3IABkFQ] |
| RPA forward primer  (30 nt) | GAACTACTGTCTTCACGCAGAAAGCGTCTA |
| RPA reverse primer  (34 nt) | TCCAGGCATTGAGCGGGTTTATCCAAGAAAGGAC |
| RPA probe  (48 nt) | Ggacccggtcgtcctggcaattccggtg[T(FAM)]A[dSpacer]  [T(BHQ1)]caccggttccgcaga-[SpacerC3] |
