# Supplementary material for: Rapid detection of hepatitis C virus using recombinase polymerase amplification
Source: PLoS One. 2022 Oct 25;17(10):e0276582. doi: 10.1371/journal.pone.0276582 (PMC9595512; doi:10.1371/journal.pone.0276582)
Supplement: S2 Table — As in S2 Fig, this uses the NCBI reference sequences for each genotype. This information is an estimate of mismatches due to random mutations, numerous subtypes, and recombinant forms that contribute to HCV genetic variability. (DOCX) [file pone.0276582.s002.docx]

|  | **RT-RPA Assay Mismatches** | | | |
| --- | --- | --- | --- | --- |
|  | RPA FP | RPA Probe | RPA RP | Total |
| GT1 | 0 | 0 | 0 | 0 |
| GT2 | 0 | 3 | 4 | 7 |
| GT3 | 3 | 5 | 5 | 13 |
| GT4 | 0 | 4 | 2 | 6 |
| GT5 | 0 | 2 | 1 | 3 |
| GT6 | 0 | 0 | 3 | 3 |
