# Supplementary material for: Rapid detection of hepatitis C virus using recombinase polymerase amplification
Source: PLoS One. 2022 Oct 25;17(10):e0276582. doi: 10.1371/journal.pone.0276582 (PMC9595512; doi:10.1371/journal.pone.0276582)
Supplement: S3 Table — (DOCX) [file pone.0276582.s003.docx]

| **Amplification Method** | **Reagent Cost per Reaction** |
| --- | --- |
| PCR (TaqMan Fast Virus 1-Step Master Mix) | $2.10 |
| LAMP (NEB WarmStart Kit) | $2.21 |
| RPA (TwistAmp Exo Kit) | $2.89 |
