# Supplementary figures and images for: Rapid detection of hepatitis C virus using recombinase polymerase amplification
Source: PLoS One. 2022 Oct 25;17(10):e0276582. doi: 10.1371/journal.pone.0276582 (PMC9595512; doi:10.1371/journal.pone.0276582)

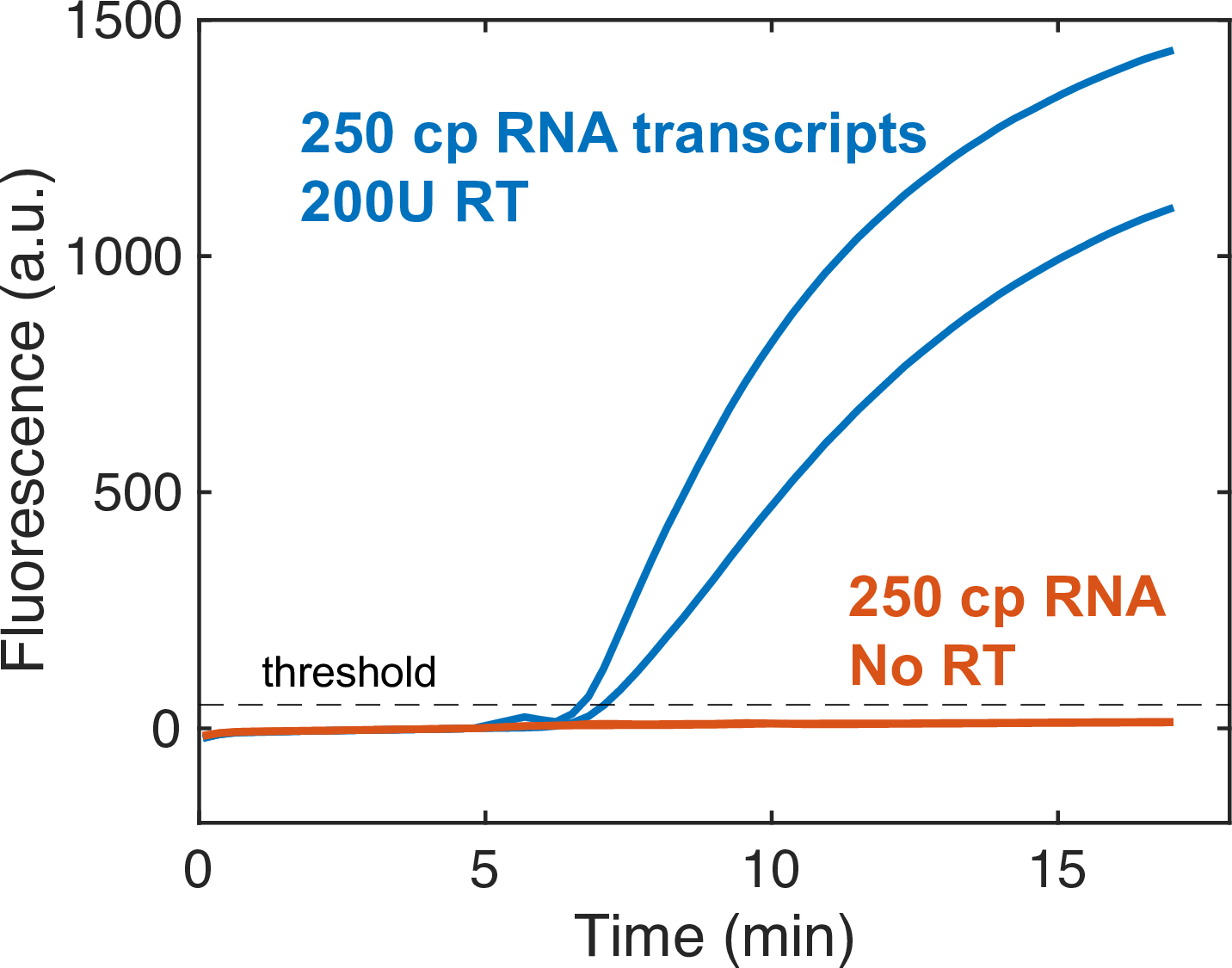

Supplement: S1 Fig — Data in blue represent RPA reactions that included both 250 copies of HCV RNA transcripts and 200 U of reverse transcriptase (N = 2). Data in red represent reactions with 250 copies of RNA transcripts but with no reverse transcriptase (N = 2). This demonstrates reverse transcriptase is needed for successful amplification, and that there are no contaminating DNA gene fragments of the HCV 5’UTR gene present in the RNA transcripts. (TIF) [file pone.0276582.s004.tif]

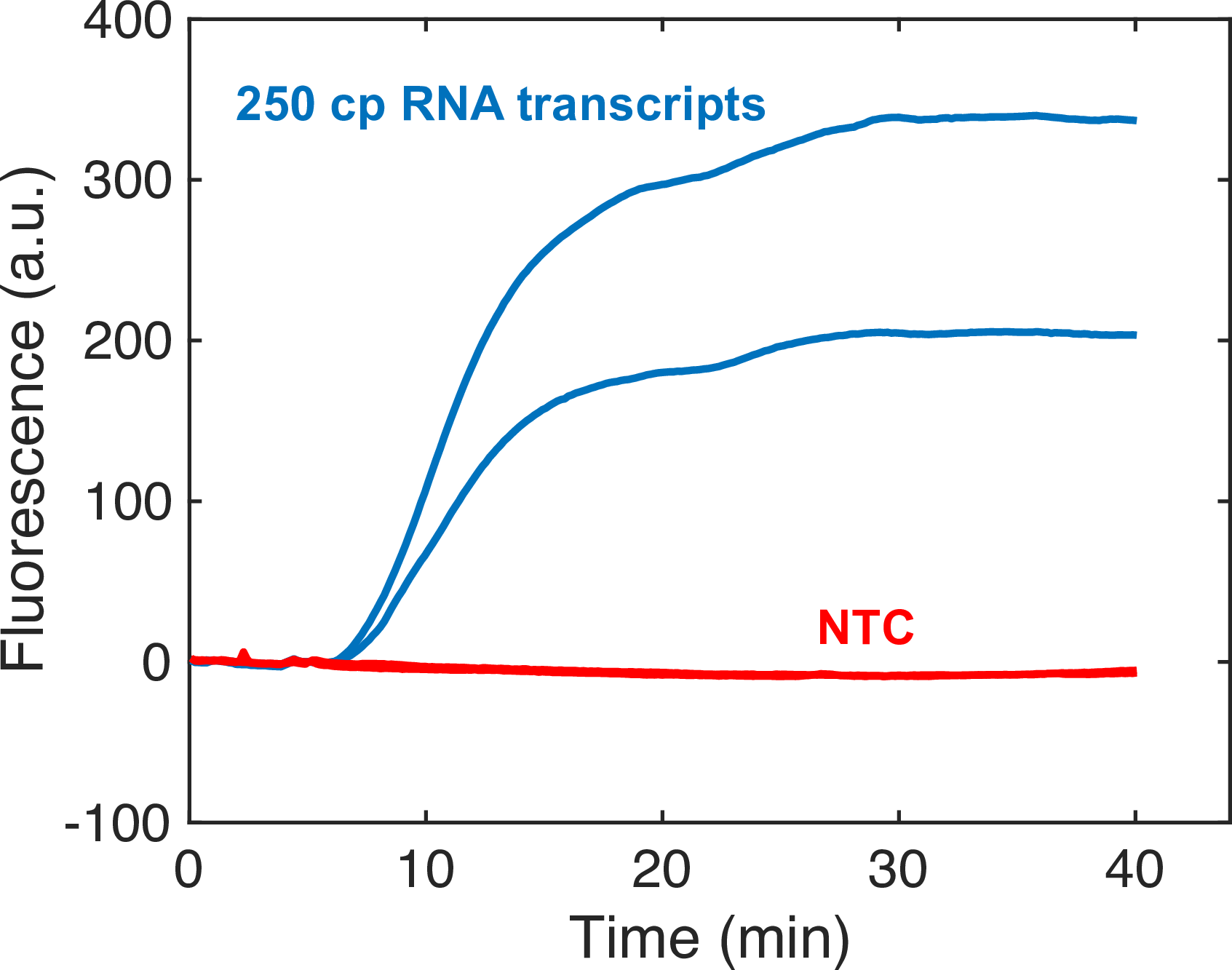

Supplement: S2 Fig — Experiments with extended incubation of RT-RPA demonstrate no template controls (NTCs) do not increase in fluorescence if incubated up to 40 minutes. Data in blue represent RPA reactions that included 250 copies of HCV RNA transcripts (N = 2), and data in red represent NTC reactions (N = 2). (TIF) [file pone.0276582.s005.tif]

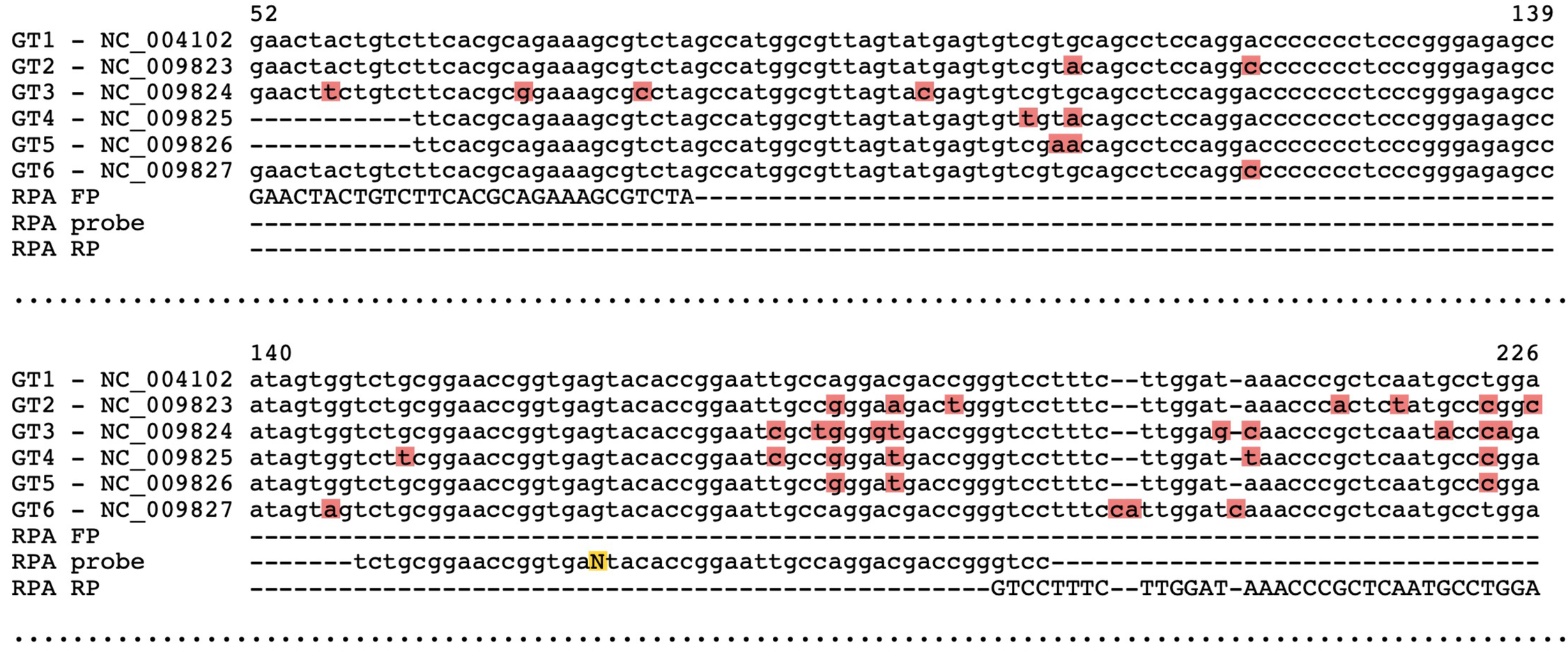

Supplement: S3 Fig — The NCBI reference sequences for each respective genotype were used here (accession numbers included in the label). Only the target region of the RPA assay is shown. Mismatches are highlighted in red with respect to the genotype 1 sequence. (TIF) [file pone.0276582.s006.tif]
